# Influence of disruption of the acromioclavicular and coracoclavicular ligaments on glenohumeral motion: a kinematic evaluation

**DOI:** 10.1186/s12891-016-1330-2

**Published:** 2016-11-17

**Authors:** Kempland C. Walley, Babak Haghpanah, Andreas Hingsammer, Ethan R. Harlow, Ashkan Vaziri, Joseph P. DeAngelis, Ara Nazarian, Arun J. Ramappa

**Affiliations:** 1 Center for Advanced Orthopaedic Studies, Carl J. Shapiro Department of Orthopaedic Surgery, Beth Israel Deaconess Medical Center, Harvard Medical School, Boston, MA USA; 2 Carl J. Shapiro Department of Orthopaedic Surgery, Beth Israel Deaconess Medical Center, Harvard Medical School, Boston, MA USA; 3 Department of Mechanical and Industrial Engineering, Northeastern University, Boston, MA USA; 4 Carl J. Shapiro Department of Orthopaedic Surgery, Beth Israel Deaconess Medical Center, 330 Brookline Avenue, Stoneman 10, Boston, MA 02215 USA

**Keywords:** Glenohumeral joint, Acromio-clavicular ligaments, Coraco-clavicular ligaments, Kinematics, Type II AC injury, Type III AC injury, Ligament resection

## Abstract

**Background:**

Changes to the integrity of the acromioclavicular (AC) joint impact scapulothoracic and clavicular kinematics. AC ligaments provide anterior-posterior stability, while the coracoclavicular (CC) ligaments provide superior-inferior stability and a restraint to scapular internal rotation. The purpose of this cadaveric study was to describe the effect of sequential AC and CC sectioning on glenohumeral (GH) kinematics during abduction (ABD) of the arm. We hypothesized that complete AC ligament insult would result in altered GH translation in the anterior-posterior plane during abduction, while subsequent sectioning of both CC ligaments would result in an increasing inferior shift in GH translation.

**Methods:**

Six cadaveric shoulders were studied to evaluate the impact of sequential sectioning of AC and CC ligaments on GH kinematics throughout an abduction motion in the coronal plane. Following an examination of the baseline, uninjured kinematics, the AC ligaments were then sectioned sequentially: (1) Anterior, (2) Inferior, (3) Posterior, and (4) Superior. Continued sectioning of CC ligamentous structures followed: the (5) trapezoid and then the (6) conoid ligaments. For each group, the GH translation and the area under the curve (AUC) were measured during abduction using an intact cadaveric shoulder. Total translation was calculated for each condition between ABD 30° and ABD 150° using the distance formula, and a univariate analysis was used to compare total translation for each axis during the different conditions.

**Results:**

GH kinematics were not altered following sequential resection of the AC ligaments. Disruption of the trapezoid resulted in significant anterior and lateral displacement of the center of GH rotation. Sectioning the conoid ligament further increased the inferior shift in GH displacement.

**Conclusion:**

A combined injury of the AC and CC ligaments significantly alters GH kinematics during abduction. Type III AC separations, result in a significant change in the shoulder’s motion and may warrant surgical reconstruction to restore normal function.

## Background

The shoulder girdle experiences great stress and strain during contact and overhead sports. Injuries to the acromioclavicular (AC) joint are often affect the coracoclavicular (CC) ligaments [1]. They account for up to 9% of all shoulder injuries and are second only to glenohumeral joint dislocations [2]. Changes to the integrity of the AC joint impact the shoulder’s function [3–5] since they alter both scapulothroacic and glenohumeral (GH) kinematics [1, 6, 7]. Understanding the AC joint’s influence on shoulder function may offer insight into how surgical techniques can optimize patient outcomes. Prior investigations have focused on glenohumeral motion in the setting of impingement, instability, and rotator cuff pathology [1, 8–12]. The complex relationships that govern the kinematics of the shoulder have been rigorously characterized through sequential sectioning of the AC and CC ligaments in cadaveric studies [13, 14]. Current literature suggests that AC ligaments provide anterior-posterior stability and axial rotation of the clavicle [13, 14]. The CC ligament complex provides superior-inferior stability and a restraint to scapular internal rotation [1, 15–17]. The quantitative contributions of the AC and CC ligaments toward GH kinematics during abduction, however, are poorly understood/ No cadaveric study has investigated this phenomenon to date.

The paucity of literature regarding the importance of the AC and CC ligaments may be related to the difficulty of designing and executing appropriate, reproducible, and physiologically relevant experiments that can test the AC and CC joints with a precise range of motion (ROM) in cadaveric models. Existing data are often affected by measurement errors due to discrete, rather than continuous, data acquisition.[18–23] Additionally, the complex interplay of the glenohumeral, scapulothoracic, and sternoclavicular joints represent a challenge to the most sophisticated investigator.

In this study, we characterized the effect of sequential sectioning of the AC ligaments, and CC ligaments using a validated, automated upper extremity testing system with seven degrees-of-freedom (DOF) and continuous data collection to describe the effect of type II and type III AC separations on the shoulder’s kinematics [24]. *We hypothesized that: a type II injury (complete injury to the AC ligaments) would result in altered GH translation in the anterior-posterior plane during abduction of the arm; and that a type III separation (injury to the AC and CC ligaments) would result in an increasing inferior shift in GH translation during abduction.*

## Methods

### Testing apparatus

**Fig. 1:**
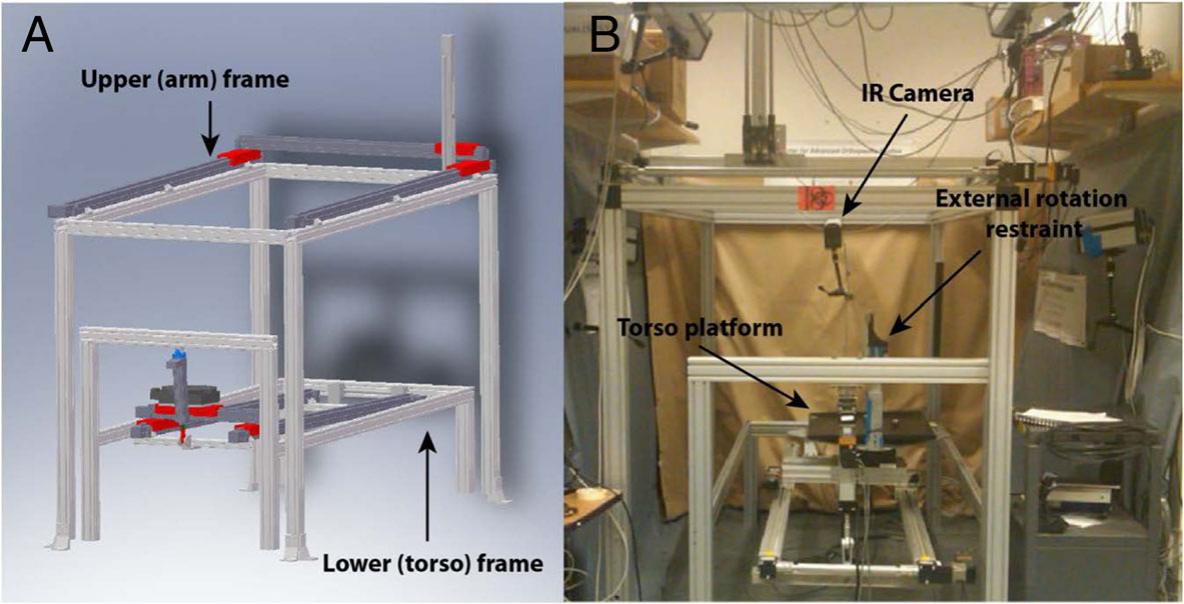
Robotic testing system that generates automated motion segments for a cadaveric torso over a designated trajectory. The seven degrees of freedom testing apparatus was designed and manufactured with four actuators on the lower frame to move the torso and with three actuators on the upper frame to move the hand with an additional rotational axis added to the lower frame to rotate the torso. **a** Apparatus schematic. **b** Apparatus photograph

An automated upper extremity testing system was used to precisely move each specimen using a prescribed motion trajectory.[24–28]. This system encompasses a lower frame (Fig. 1a), which houses an intact cadaveric torso, and an upper frame, which controls the upper extremity to affect a programmed motion trajectory (Fig. 1b). The torso frame allows translation in the x-, y-, and z-axes as well as rotation around the z-axis. The upper extremity frame allows for translation of a specimen’s arm in in the x-, y-, and z-axes. All seven degrees-of-freedom (DOF) are controlled using actuators via a centralized controller. Programmable software generates a precise motion trajectory reproducibly and accurately within the limits of the actuators. The coefficient of variation is less than 0.5% for all axes. The absolute and percent errors in the displacement of all axes were 0.1 and 0.5%, respectively^10^.

### Cadaveric specimens

Fresh-frozen human cadaveric torsos were acquired from Medcure Anatomical Tissue Bank (Portland, Oregon, USA). Three torsos from Caucasian males with an average age of 55 ± 4 years, height of 190 ± 4 cm, and body mass index (BMI) of 27.1 ± 1.85 kg/m^2^ were used for this study. Both shoulders were tested on each specimen for a total of six shoulders. Torsos were mounted on a rod and foam fixture, as previously described, and a Schanz pin was inserted through the distal radius and ulna after the hand was disarticulated [26]. For all specimens, the trunk was stabilized to the torso frame and the scapula was allowed to move as the arm articulated. For each shoulder, the skin and the deltoid muscle were removed [27]. Retro-reflective marker clusters were placed in the humeral shaft, the postero-lateral acromion, and the sternum [25, 27].

### Motion analysis

Kinematic data were acquired by recording the motion of the retro-reflective marker clusters using five Qualisys Pro Reflex 120 Hz (Qualisys AB, Göteborg, Sweden) cameras (Fig. 1b). [26] A multi-aspect calibration was performed to define the volumetric testing space and to characterize each specimen’s anatomy following guidelines established by the International Society of Biomechanics [29]. When fully calibrated, the system can detect movements greater than or equal to 0.3 mm. The motion of each segment and the instant center of rotation of the GH joint (COR_GH_) were calculated relative to the scapular reference frame [30]. The x-, y-, and z-axes corresponded to anterior-posterior (AP), superior-inferior (SI), and medial-lateral (ML) planes, respectively.

### Simulation of abduction and implementation of sequential sectioning of AC and CC ligaments

During testing, the arm was abducted from 30° (ABD 30) to 150° (ABD 150) at a constant speed in the coronal plane. Throughout this motion trajectory, the humerus was held in neutral rotation. All abductions were passively simulated.

Each specimen served as its own internal control. Changes in GH kinematics were reported relative to the preceding condition to limit the effect of hysteresis and anatomic variation. All conditions were reported as the average of three repetitions.

**Fig. 2:**
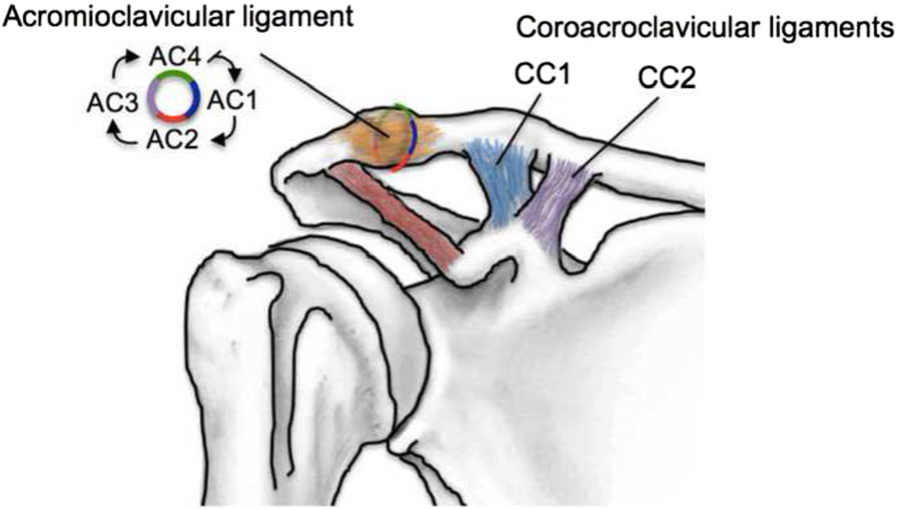
The AC ligaments were then sectioned sequentially according to the following order: Anterior (AC1), Inferior (AC2), Posterior (AC3), and Superior (AC4) ligaments. Following the division of the AC ligaments, the CC ligaments were sectioned in two steps. First, the trapezoid ligament (CC1) was sharply incised, and then the conoid ligament (CC2) was cut. After each step, the specimen was abducted three times

To establish a baseline (BL), each shoulder was abducted three times from 30° to 150° in the native state. The AC ligaments were then sectioned sequentially: (1) Anterior, (2) Inferior, (3) Posterior, and (4) Superior. After each step, the specimen was abducted three times. The CC ligaments were sectioned in two steps. First, the trapezoid was sharply incised followed by the conoid ligament (Fig. 2). Each specimen was subjected to the same abduction motion a total of three times following each ligament sectioning.

### Statistical analysis

Motion was recorded continuously from ABD 30° to ABD 150°. For each condition, the average translation was plotted over time to calculate the total translation and the area under the curve (AUC) during each motion segment. The absolute GH translation was calculated for each step of the sequential sectioning (BL, Anterior AC, Inferior AC, Posterior AC, Superior AC, Trapezoid, and Conoid). A generalized estimating equations analysis (GEE) was performed to compare GH translation on each axis. The AUC was calculated for each condition on each axis by use of the trapezoidal rule to appropriately assess the path-dependent motion (Matlab v12; MathWorks, Natick, MA, USA). The Wilcoxon signed rank test was used to compare the AUCs among the conditions. Total translation was calculated for each condition between ABD 30° and ABD 150° using the distance formula, and a univariate analysis of variance (ANOVA) was used to compare total translation for each axis during the different conditions. Statistical analysis was conducted with IBM SPSS (v21.0; IBM-SPSS Inc., Armonk, New York, USA). Two-tailed P values less than 0.05 were considered significant.

With six specimens from three donors included (three pairs), a statistical power of 80% allowed for detection of a difference of greater than 1.0 mm of GH translation between the different conditions and 85% power to detect mean differences of greater than 1.2 mm of translation using ANOVA with a compound symmetry correlation structure to handle the paired specimens (nQuery Advisor, Statistical Solutions, Boston, MA, USA).

## Results

**Fig. 3:**
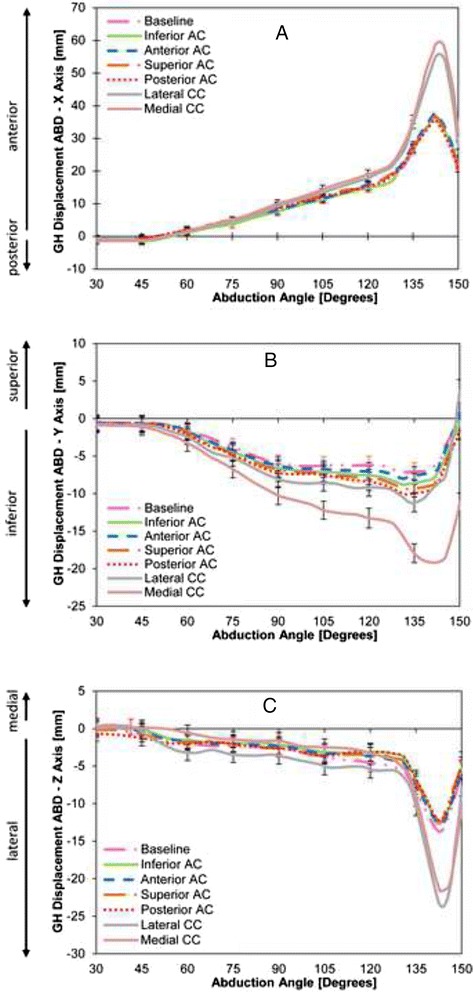
Glenohumeral joint translation in Anterior-Posterior (**a**), Superior-Inferior (**b**), and Medial-Lateral (**c**) planes for all conditions of sequential sectioning of AC and CC ligaments. * denotes significant difference

In the anterior-posterior (AP) plane, there were no differences in translation of the center of rotation of the GH head, with respect to the glenoid, following complete resection of the AC ligaments (all sequential AC ligamentous conditions, for all angles *p* > 0.05). Trapezoid resection resulted in a significant increase in anterior displacement of the center of rotation of the GH head beyond 130° ABD (*p* < 0.05; Fig. 3a). This anterior shift measured approximately 20 mm at its maximum (140°). Sectioning the conoid ligament did not result in an additional increase in the GH displacement (*p* > 0.05 for all angles; Fig. 3a).

In the superior-inferior (SI) plane, no differences in the translation of the center of rotation of the GH head were found following sectioning of all AC ligaments (for all angles *p* > 0.05). Trapezoid resection *did not* result in a significant change either. However, resection of the conoid ligament resulted in a significant inferior shift in GH displacement beyond that achieved from sectioning the trapezoid (*p* < 0.05; Fig. 3b). This suggests that a complete rupture of the CC ligaments (trapezoid *and* conoid) significantly alters GH kinematics in the SI plane. The maximum inferior shift measured approximately 10 mm at 140° ABD.

In the medial-lateral (ML) plane, no differences in translation of the center of rotation of the GH head with respect to the glenoid were found following resection of the AC ligaments (all sequential AC ligamentous conditions, for all angles *p* > 0.05; Fig. 3c). Trapezoid resection caused a significant lateral shift in the center of rotation beyond 140° ABD and throughout the motion (*p* < 0.05; Fig. 3c). This lateral shift measured approximately 10 mm at 140° ABD. No additional change was observed in GH displacement in the ML plane when the conoid ligament resected (*p* > 0.05 for all angles; Fig. 3c).

**Fig. 4:**
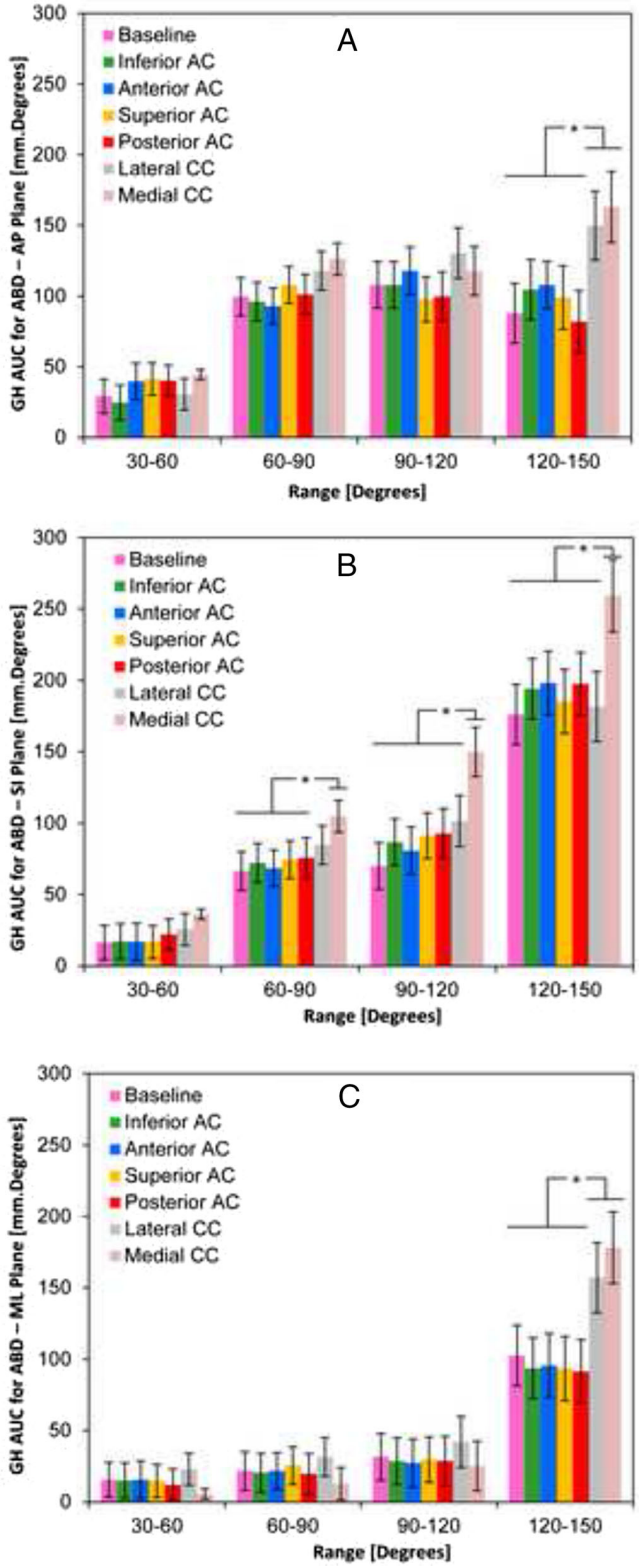
Area under the curve (AUC) analysis in Anterior-Posterior (**a**), Superior-Inferior (**b**), and Medial-Lateral (**c**) planes for all conditions of sequential sectioning of AC and CC ligaments. * denotes significant difference

The area under the curve of GH translation was compared to assess the path-dependence of the translation motion throughout abduction. The AUC analysis revealed that trapezoid sectioning affected the motion trajectory in the AP and ML planes following 120° of abduction. Further sectioning of the conoid significantly altered the motion trajectory in the SI planes in abduction angles greater then 60° (*P* ≤ 0.05; Fig. 4a and b). The AUC analysis also revealed that complete loss of CC ligaments had a significant effect on the motion trajectory in the ML plane above 60° of abduction (*P* ≤ 0.05; Fig. 4c).

## Discussion

In this investigation, the trapezoid ligament provided a restraint to both *anterior* and *lateral* glenohumeral translation beyond 130° and 140° of abduction, respectively (Fig. 4a and c). In the superior-inferior plane, the conoid ligament influenced glenohumeral kinematics beyond 90° of abduction, providing a restraint to *inferior* glenohumeral translation (Fig. 4b). These results suggest that the CC ligaments stabilize the GH joint in the horizontal and vertical planes. Interestingly, the passive effect of the CC ligaments limits the upward rotation of the scapula at the AC joint [31], and they prevent upward tilting of the glenoid fossa during abduction greater than 90° [7]. Beyond 90° ABD, the loss of CC ligaments uncouples scapular abduction and upward rotation, altering GH kinematics by forward flexing of the scapula.

The importance of the AC and CC ligaments in acromioclavicular, glenohumeral, and scapulothoracic motion is broadly acknowledged [1, 13, 32–34]. Through serial sectioning of the AC and CC ligaments, Urist concluded that the AC ligaments were essential stabilizers of distal clavicle [35]. It can be dislocated anteriorly and posteriorly after sectioning of the AC ligaments. However, superior translation of the clavicle is only possible following the division of the CC ligaments. When the CC ligaments are completely detached, the shoulder girdle displaces downward and the clavicle moves superiorly by the secondary action of the trapezius. This in-vitro understanding is supported by the work of Rockwood et al., Fukuda et al., Lee et al., Skjeldal et al., Branch et al., and Flatow et al. [14, 16, 17, 36–39]. The AC ligaments resist clavicular translation in the horizontal plane, while the CC ligaments prevent vertical displacement. Anatomic descriptions of the superior AC capsule reveal that it is thickest posteriorly to limit clavicular translation in the horizontal plane [33, 40–43].

Similarly, the CC ligaments are known to influence scapular kinematics. Satoshi et al. reported that sectioning the CC ligaments caused scapular internal rotation and loss of coordination between posterior scapular tilting and posterior clavicular rotation [7]. These results demonstrate the important role of the CC ligaments in maintaining full ROM during elevation of the upper extremity.

Klimkiewicz et al. investigated the relative contribution of the individual AC ligaments in inhibiting posterior translation of the distal clavicle [13]. Sectioning of the anterior and inferior AC ligaments did significantly affect AP translation of the distal clavicle. However, sectioning of the superior and posterior AC ligaments had a pronounced effect. Interestingly, the superior AC ligament was the strongest, providing 56% of the resistance to translation in the horizontal plane, while the posterior ligament provided 25%[13]. In this way, the superior and posterior AC ligaments are the most important contributors to the AC joint stability when preventing posterior clavicular translation. These findings, however, must be considered in the context of their experimental limitations and simplifications. Serial sectioning was performed on isolated specimens, ignoring the effect of the glenohumeral and scapulothoracic articulation. Additionally, when a bi-planar analysis was used, the analysis of shoulder kinematics may be oversimplified.

While prior cadaveric studies have described changes in scapular and clavicular kinematics following sequential sectioning of the AC and CC ligaments, these changes have not been described for the GH joint. Considering the work of Klimkiewicz et al.[13], we hypothesized that serial sectioning of the AC joint would alter GH motion in all planes of motion, and that effect would be most prominent following the division of the superior and posterior AC ligaments. Correspondingly, we hypothesized that subsequent sectioning of the CC ligaments would further alter GH motion.

From a clinical perspective, our findings support the current opinion regarding the management of type II and type III AC joint injuries [13]. For a type II AC separation, non-operative treatment is recommended because this pattern of injury results in little change in GH kinematics. For type III AC separations, there is a complete rupture of both the AC and CC ligaments. In these patients, operative treatment may be indicated because the shoulder kinematics are significantly affected. Reconstruction of the AC and CC ligaments aims to restore the normal motion of the shoulder girdle.

Our study should be interpreted in light of the inherent limitations of the findings. As a cadaveric model, the collected data represent the passive motion of the shoulder during abduction and do not simulate the dynamic forces essential to glenohumeral stability [43]. Additionally, we tested each specimen in abduction only and did not include flexion, extension, adduction, internal rotation or external rotation. Further assessment of these motions could reveal more alternative behavior. Continued research in this space warrants consideration.

The precision of our measurements depends upon the accuracy of the anatomical landmark calibration. Errors may be introduced by GH translation through the regression analysis used to calculate the instant center of rotation [25, 26], and variability in data could result from the nature of anatomical landmarks as areas rather than discrete points [44]. Another consideration is the speed of the abduction motion. This simulation was performed at a speed slower than normally performed during daily living. Bergmann et al. have shown that reducing the speed of a specific upper extremity motion may change GH peak forces and corresponding moments [45]. However, the direction of the GH forces remains constant [45]. In this study, the deltoid was removed [26]. While it has been suggested that the deltoid may reduce GH translation due to its bulk effect [46], in this study, each shoulder specimen functioned as its own internal control. Thus, our analysis does not report the absolute GH translation, rather the relative changes between different testing conditions (BL, AC1, AC2, AC3, AC4, CC1, CC2). We report significant differences in translation and area under the curve for GH translation relative to arm position. The testing apparatus is computer-controlled, offering highly precise and accurate identification of subtle difference in GH kinematics.

## Conclusions

In this kinematic study of the cadaveric shoulder, combined AC and CC ligament injuries significantly alter GH kinematics during abduction. It was demonstrated that AC ligament ruptures alone (type II AC separation) did not result in altered GH translation during abduction. When the CC ligaments were also compromised (type V AC separation), there was a significant change in GH kinematics. The trapezoid ligament provides a restraint to both anterior and lateral GH translation beyond 130° and 140° of abduction, respectively, while the conoid ligament influences GH motion beyond 90° of abduction in the superior-inferior plane. It provides a restraint to inferior GH translation. In patients with type III AC separations, surgical reconstruction of the AC and CC ligaments may improve shoulder function by normalizing GH motion.

## Abbreviations

ABD: Abduction
AC: Acromioclavicular
ANOVA: Univariate analysis of variance
AP: Anterior-posterior
AUC: Area under the curve
BL: Baseline
BMI: Body mass index
CC: Coracoclavicular
COR_GH_: Center of rotation of the GH joint
GH: Glenohumeral
ML: Medial-lateral
SI: Superior-inferior
